# Grapefruit Derived Flavonoid Naringin Improves Ketoacidosis and Lipid Peroxidation in Type 1 Diabetes Rat Model

**DOI:** 10.1371/journal.pone.0153241

**Published:** 2016-04-13

**Authors:** Alfred N. Murunga, David O. Miruka, Christine Driver, Fezile S. Nkomo, Snazo Z. Z. Cobongela, Peter M. O. Owira

**Affiliations:** Molecular and Clinical Pharmacology Research Laboratory, Department of Pharmacology, Discipline of Pharmaceutical Sciences, School of Health Sciences, University of KwaZulu-Natal, P.O. Box X5401, Durban, South Africa; Broad Institute of Harvard and MIT, UNITED STATES

## Abstract

**Background:**

Hypoglycemic effects of grapefruit juice are well known but the effects of naringin, its main flavonoid on glucose intolerance and metabolic complications in type 1 diabetes are not known.

**Objectives:**

To investigate the effects of naringin on glucose intolerance, oxidative stress and ketonemia in type 1 diabetic rats.

**Methods:**

Sprague-Dawley rats divided into 5 groups (n = 7) were orally treated daily with 3.0 ml/kg body weight (BW)/day of distilled water (group 1) or 50 mg/kg BW of naringin (groups 2 and 4, respectively). Groups 3, 4 and 5 were given a single intra-peritoneal injection of 60 mg/kg BW of streptozotocin to induce diabetes. Group 3 was further treated with subcutaneous insulin (4.0 IU/kg BW) twice daily, respectively.

**Results:**

Stretozotocin (STZ) only-treated groups exhibited hyperglycemia, polydipsia, polyuria, weight loss, glucose intolerance, low fasting plasma insulin and reduced hepatic glycogen content compared to the control group. Furthermore they had significantly elevated Malondialdehyde (MDA), acetoacetate, β-hydroxybutyrate, anion gap and significantly reduced blood pH and plasma bicarbonate compared to the control group. Naringin treatment significantly improved Fasting Plasma Insulin (FPI), hepatic glycogen content, malondialdehyde, β-hydroxybutyrate, acetoacetate, bicarbonate, blood pH and anion gap but not Fasting Blood Glucose (FBG) compared to the STZ only-treated group.

**Conclusions:**

Naringin is not hypoglycemic but ameliorates ketoacidosis and oxidative stress. Naringin supplements could therefore mitigate complications of diabetic ketoacidosis.

## Introduction

Diabetes mellitus (DM) is a consequence or absolute of relative insulin deficiency leading to hyperglycemia and concomitant disturbances in carbohydrate, fat and protein metabolism [1, 2].

Diabetic ketoacidosis (DKA) is an acute life threatening complication of DM. It is defined by blood glucose >11 mmol/l, venous pH <7.3, and bicarbonate <15 mM, ketonemia and ketonuria [3, 4]. DKA primarily affects patients with type 1 but can also occur in type 2 diabetes under conditions of metabolic stress such as infection, trauma and surgery [5].

Hyperglycemia-induced oxidative stress causes pancreatic β-cell dysfunction due to pro-inflammatory cytokines which induce the release of insulin counter-regulatory hormones (glucagon, cortisol and growth hormone) leading to increased hepatic gluconeogenesis and hyperglycemia [6, 7, 8]. Increased lipolysis accelerate the delivery of free fatty acids to the liver for ketone body {acetoacetate (AcAc) and β-hydroxybutyrate (3-HB)} synthesis [7, 9]. AcAc and 3-HB are strong organic anions that dissociate freely generating increased hydrogen ions which overwhelm the normal plasma bicarbonate buffering capacity resulting in metabolic acidosis and increased anion gap (AG), (defined as the sum of serum chloride and bicarbonate concentrations subtracted from the serum sodium concentrations) [9, 10, 11].

Therapeutic management of DKA is yet to be optimised but includes adequate fluid replacement and insulin infusion to correct electrolyte imbalance and hyperglycaemia, respectively [4]. Currently, there are no clear-cut guidelines defining loss of glycemic control or propensity to hyperosmolar states (blood glucose of 33 mM or more) which may predispose vulnerable patients to DKA. However, monitoring of sodium, potassium, magnesium and phosphorus levels to maintain electrolyte balance, hemogram to assess anaemia and blood pH and gas analysis to determine ketonemia are routinely done. Contentious issues on fluid replacement therapy include the amount and type of fluids (normal saline or Ringer’s lactate) to be used and the rate of delivery [4, 12]. Routinely, normal saline is used for fluid expansion followed by intravenous insulin infusion at 0.1 U/kg/h until the patient is stabilised with dextrose to maintain euglycemia then switched to subcutaneous insulin with a dietary plan [12]. However, hospitalisation, stabilisation and subsequent follow-up pose challenges especially to patients with disadvantaged socio-economic backgrounds hence a dietary plan that mitigates the onset of DKA may be a viable cost effective patient care option.

Consequently, medicinal plants which have traditionally been used to manage diabetes offer some hope as they have less side-effects commonly associated with conventional medications [9]. Naringin (4’,5,7-trihydroxy flavonone-7-rhamnoglucoside), the major flavonoid in grapefruit juices has been shown to possess pharmacological properties such as antioxidant, antidiabetic and antidyslipidemic effects [13, 14, 15, 16]. Hypoglycemic effects of naringin are well documented [17, 18] and Punithavathi et al [15] have further shown that 30 mg/kg of naringin co-administered with 50 mg/kg of vitamin C prevented oxidative stress, improved fasting plasma insulin concentrations and glucose intolerance. Hyperglycemia in a diabetic state is associated with increased oxidative stress [15, 16] which exacerbates DKA hence it is envisaged that with its demonstrated antioxidant effects, naringin could ameliorate glucose intolerance and metabolic complications associated with DKA. This study was designed to investigate the effects of naringin on glucose intolerance, metabolic benchmarks of DKA and oxidative stress in streptozotocin (STZ)-induced diabetes in rats.

## Materials and Methods

### Chemicals and Reagents

Naringin, D-glucose, STZ, citrate buffer, hydrochloric acid, sulphuric acid, potassium hydroxide, ethanol, sodium sulphate and phenol were all purchased from Sigma-Aldrich Pty. Ltd, Johannesburg, South Africa.

Insulin (Novo Nordisk®, Norway), normal saline, portable glucometers and glucose test strips (Ascencia Elite™, Bayer Leverkusen, Germany) were purchased from a local pharmacy. Halothane and other accessories were provided by the Biomedical Resource Unit (BRU) of the University of KwaZulu-Natal, Durban, South Africa.

### Animal Treatment

Male Sprague-Dawley rats of 200-300g body weight were provided by the Biomedical Resource Unit of the University of KwaZulu-Natal and divided into 5 groups (n = 7), housed seven rats per cage. Animals were given free access to standard commercial chow and drinking tap water *ad libitum*. The rats were maintained on a 12 hour dark-to-light cycle of 08.00 to 20.00 hours light in an air controlled room (temperature 25 ±2°C, humidity 55% ±5%) and were handled humanely, according to the guidelines of the Animal Ethics Committee of University of KwaZulu-Natal which approved the study.

Type 1 DM was induced by a single intraperitoneal injection of 60 mg/kg BW of STZ dissolved in 0.2 ml citrate buffer, pH 4.5 after an overnight fast in groups 3, 4 and 5, respectively. Diabetic status was confirmed 3 days after STZ administration by tail pricking to analyse the blood glucose levels. Animals with random blood glucose concentrations above 11.0 mmol/L were considered diabetic and were included in the study [19] and immediately commenced on treatment.

Naringin (50 mg/kg BW/day in distilled water) was orally administered to groups 2 and 4 while regular insulin (4.0 IU/kg) was further administered subcutaneously twice daily to group 3. Distilled water (3.0 ml/kg BW/day) was administered to groups 1 and 5, respectively orally, (Table 1). Animal weights and water consumption were measured daily. The animals were further placed in solitary metabolic cages on day 40 of treatment and 24-hour urine samples collected measured and recorded. The animals were sacrificed by halothane overdose on day 42 of treatment and blood samples collected via cardiac puncture and plasma samples stored at -80°C for further biochemical analysis.

**Table 1:** Animal treatment protocol. The animals were weighed and randomly divided into 5 groups, (n = 7). Naringin (mg/kg BW, orally), insulin (IU/kg BW, SC) and distilled water (mls/kg BW, orally) were orally administered daily. BW = per kg body weight.

Groups	Normal control	Normal naringin	STZ-insulin	STZ-Naringin	STZ
1	3.0				
2		50.0			
3			4.0		
4				50.0	
5					3.0

### Blood Glucose Testing

FBG measurements were done on treatment days 0, 14 and 28, respectively. On treatment day 41, Glucose Tolerance Tests (GTT) were done after an overnight fast followed by a single intraperitoneal injection of 3.0 mg/kg BW of glucose in normal saline and blood glucose levels were measured at times 0, 15, 30, 60, 90 and 120 minutes. Insulin treatment for group 4 was withheld on the day of GTT. Blood glucose analysis was done as previously described and the calculated Area under the curve (AUC) from blood-glucose concentration-time curves presented as AUC units {(mmol/L) X Time (minutes)}.

### Determination of Plasma Insulin

An ultra-sensitive rat insulin enzyme-linked immunoassay kit (DRG Diagnostics, Marburg, Germany) was used to analyse the plasma insulin levels as per the manufacturer’s instructions. The optical density was determined by a microplate reader (EZ Read 400, Biochrom®) at 450 nm.

### Hepatic Glycogen Assay

Hepatic glycogen content was measured by the modified method of Seifter *et al*.,[20]. Briefly, the liver tissue was homogenised in 1.0 ml of 30% KOH saturated with Na_2_SO4. The homogenate obtained was dissolved by boiling in a water bath (100˚C) for 30 minutes, vortexed and cooled on ice. Glycogen was then precipitated with 2.0 ml of 95% ethanol, vortexed, incubated on ice for 30 min and later centrifuged at 550 g for 30 min. The glycogen pellets obtained were then re-dissolved in 1.0 ml of distilled water which was thereafter treated with 1.0 ml of 5% phenol and 5 ml of 96–98% sulphuric acid respectively. This was incubated on ice bath for 30 min and the absorbance measured at 490 nm using a spectrophotometer (Genesys 20, Thermo Spectronic®). Glycogen content was expressed as mg/g liver protein.

### Determination of Serum Electrolyte and Blood pH Levels

Serum sodium (Na⁺), potassium (K⁺), chloride (Cl⁻) and bicarbonate (HC0_3_⁻) levels were analysed using an automated chemistry analyser (Beckman Coulter, Synchron LX20 Clinical Systems, California, USA) while blood pH was determined on heparinised blood using a pH/blood gas analyser (Chiron Diagnostics, Halstead, Essex, UK).

The anion gap (AG) was calculated using the formula [11]:

$$AG=\{\left[Na^{+}]+[K^{+}\right]\}-\{[Cl^{-}]+[HC0^{3-}]$$

### Plasma Thiobarbituric Acid Reactive Substances (TBARS) Assay

Plasma TBARS assay was carried out according to the modified method of Phulukdaree *et al* [21]). Briefly, 200 μl of plasma samples were added to 500 μl of 2% phosphoric acid (H_3_PO_4_), 400 μl of 7% H_3_PO_4_ and 400 μl of BHT/TBA solution in a set of clean glass test-tubes. In another set of eight clean fresh test tubes, 200 μl of serially diluted malondialdehyde (MDA) standard was added to 500 μl of 2% H_3_PO_4_, 400 μl of 7% H_3_PO_4_ and 400 μl of butylated hydroxytoluene (BHT)/ Thiobarbituric acid (TBA) solution. The reactions in both sets of tubes were initiated with 200 μl of 1.0 M HCl. All tubes were incubated in a shaking boiling water bath (100°C) for 15 minutes and cooled at room temperature. n-butanol (1.5 ml) was then added to each tube and mixed thoroughly. Top phase (200 μl) was then transferred to a 96-well micro-plate in and read at 532 and 600 nm using micro-plate reader (Spectrostar^®^). Plasma MDA concentrations were calculated using an extinction coefficient of 1.56x10^5^M^-1^cm^-1^.

### Analysis of Serum Ketone Body Concentrations

Serum and urine ketone body levels were determined by the spectrophotometric enzymatic assay kit (Enzychrom ^TM^, BioAssay systems, EKBD-100) according to the manufacturer’s instructions. The optical density (OD) was read at 340 nm using a spectrophotometer (Genesys 20, Thermospectronic) and the concentrations calculated according to the formula:

$$[AcAc]=\frac{OD\;blank-OD\;sample}{OD\;water-OD\;standard}$$

A similar procedure was repeated for the 3-β hydroxybutyrate (3-HB) assay using the 3-HB buffer, reagent and standard, respectively and the concentrations calculated using the formula:

$$[3HB]=\frac{OD\;sample-OD\;blank}{OD\;standard-OD\;water}$$

### Statistics

The data was presented as mean ± SD and analyzed by GraphPad Prism Software^®^ Version 5.0. One-Way ANOVA or Student t-tests and non-parametric Mann–Whitney tests were used where applicable to determine statistical significance. Values of P< 0.05 were taken to imply statistical significance.

## Results

### Animal Growth Change during Treatment Period

**Fig 1:**
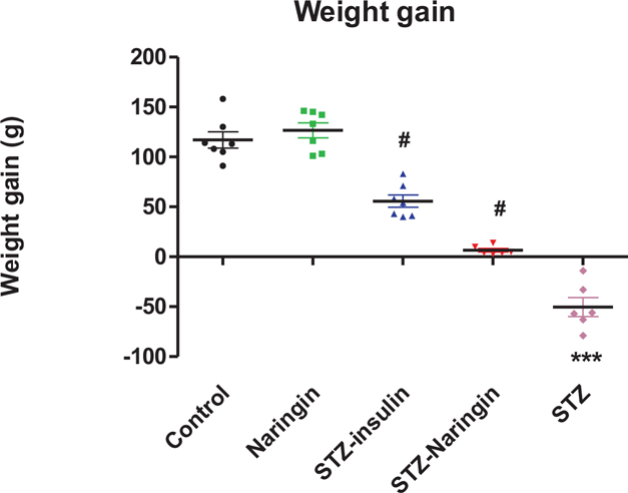
Animal weight changes during treatment period. (***p<0.0001 compared to normal control, #p<0.0001 compared to non-treated STZ group).

STZ only-treated groups exhibited significant (p<0.0001) weight loss compared to the normal control group. Treatment with insulin or naringin significantly (p<0.0001) increased weight gain compared to the non-treated STZ group. Treatment with naringin had no significant change in weight gain in normal rats compared to the control (Fig 1).

### Water Consumption during Treatment Period

**Fig 2:**
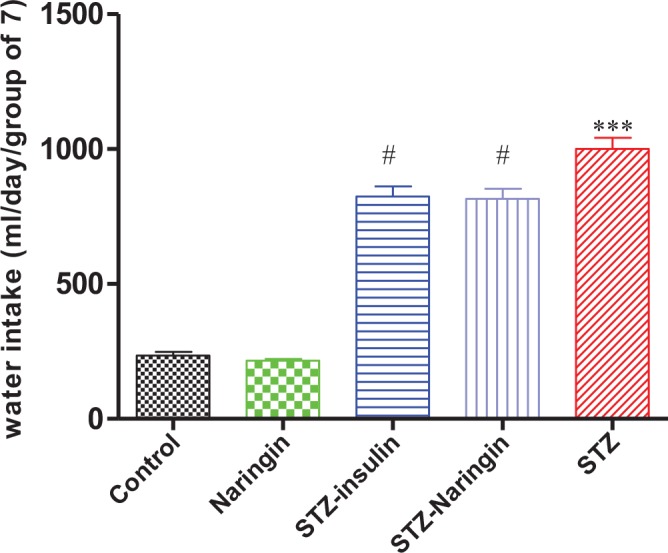
Water consumption during treatment period. (***p<0.0001 compared to normal control, #p<0.0001 compared to untreated STZ group).

The average daily water consumption was significantly (p<0.0001) higher in untreated STZ rats compared to normal control group. However, water intake was significantly (p<0.0001) reduced in the insulin or naringin treated groups compared to the non-treated STZ group (Fig 2).

### Urine Output

**Fig 3:**
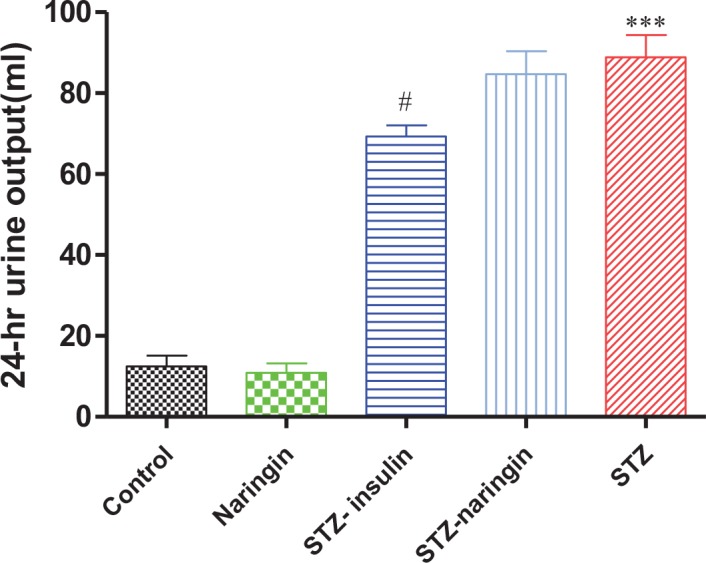
24-hour urine output collected after the animals were put in metabolic cages. (***p<0.0001 compared to normal control, # p<0.05 compared to untreated STZ group).

The urine output was significantly (p<0.0001) elevated in the untreated STZ group in comparison to the normal control group. Treatment with insulin significantly (p<0.05) reduced urine output in STZ treated animals compared to the untreated STZ group. However, naringin treatment did not have any significant effect on urine output in STZ-treated animals compared to the untreated STZ group (Fig 3).

### Glucose Tolerance

**Fig 4:**
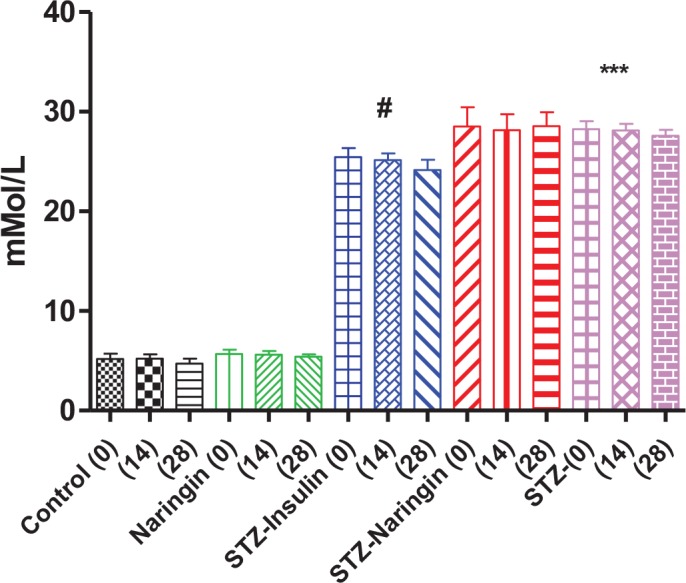
Fasting blood glucose levels measured by tail pricking after an overnight fast. (***p<0.001 compared to the normal control group, #p<0.05 compared to untreated STZ group).

FBG levels were significantly higher (p<0.0001) in the untreated STZ group compared to the normal control group. However, insulin but not naringin treatment significantly (p<0.05) improved the FBG levels compared to the untreated STZ group (Fig 4).

**Fig 5:**
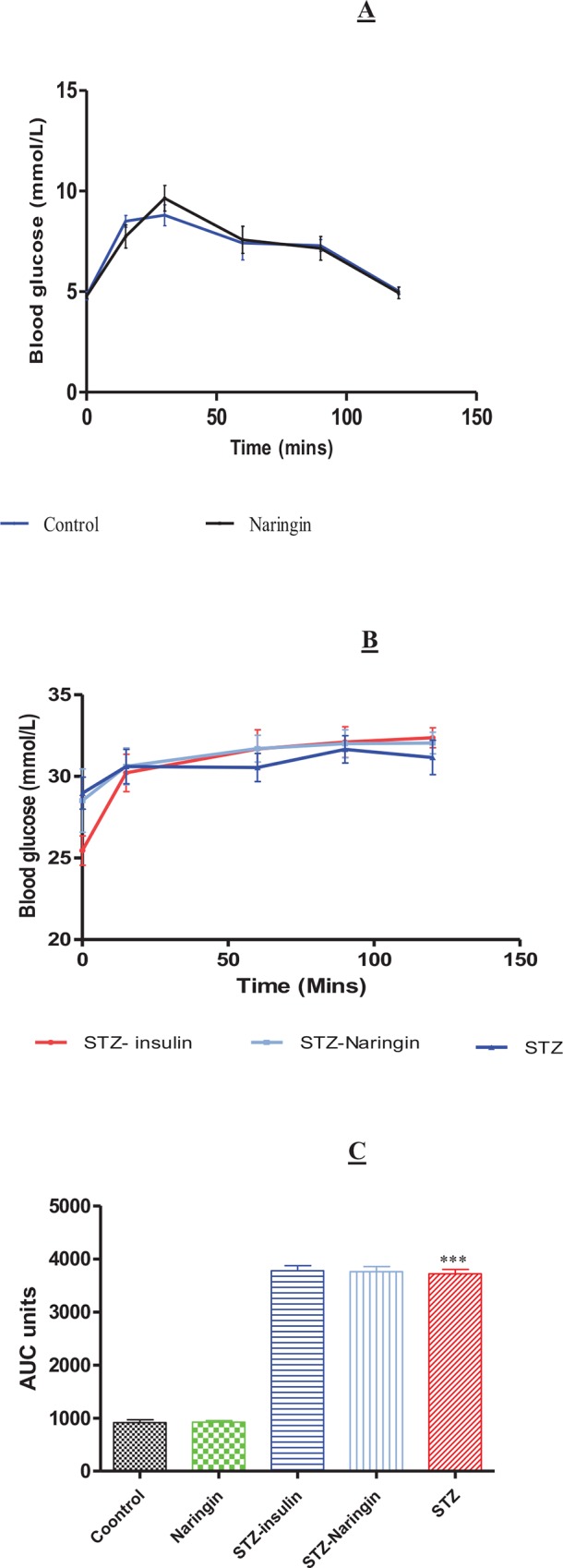
Glucose Tolerance Tests (GTT) after the animals were fasted overnight and challenged with intreperitoneal injection of 3.0 g/kg BW of glucose in normal saline in normal animals treated with distilled water (controls) or naringin (**A**) and STZ-treated animals (**B**). Insulin treatment was withheld on the day of GTT and the figures were plotted in different graphs for clarity considering the large differences in blood glucose concentrations between normal and STZ groups on the y-axis. **C**; Calculated AUC from GTT. (***p<0.0001 compared to the normal control group).

The untreated STZ group showed glucose intolerance compared to the normal control (Fig 5A and 5B) with significantly (p<0.0001) increased calculated Area Under the Curve (AUC) further compared to normal control group. Neither naringin nor insulin treatment significantly improved the AUCs in STZ-treated group (Fig 5C).

### Fasting Plasma Insulin

**Fig 6:**
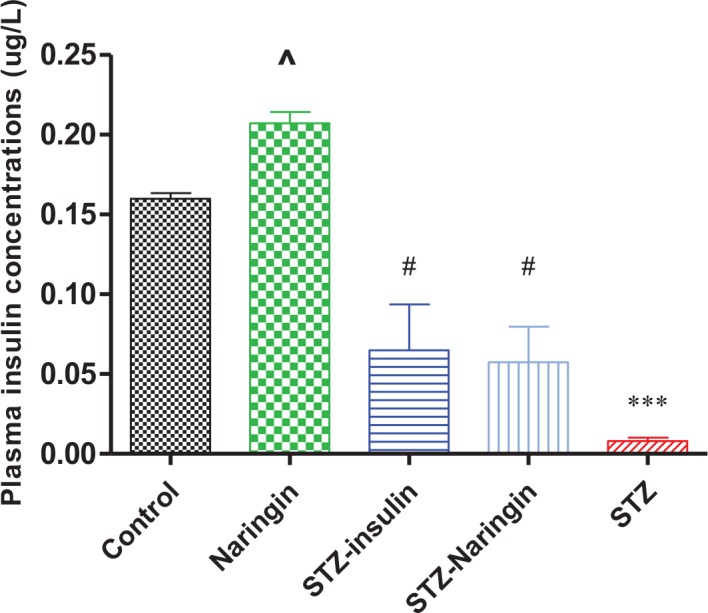
Plasma insulin concentrations after blood was collected by cardiac puncture. (***p<0.0001, ^p<0.05 compared to normal control, #p<0.05 compared to STZ only-treated animals).

FPI concentration were significantly (p<0.0001) lower in the STZ-treated group compared to the normal control group. Naringin or insulin significantly (P<0.01) improved FPI concentrations in the STZ-treated compared to the untreated STZ group, respectively (Fig 6). Surprisingly, naringin treatment significantly increased FPI concentrations in normal rats compared to the controls.

### Hepatic Glycogen Levels

**Fig 7:**
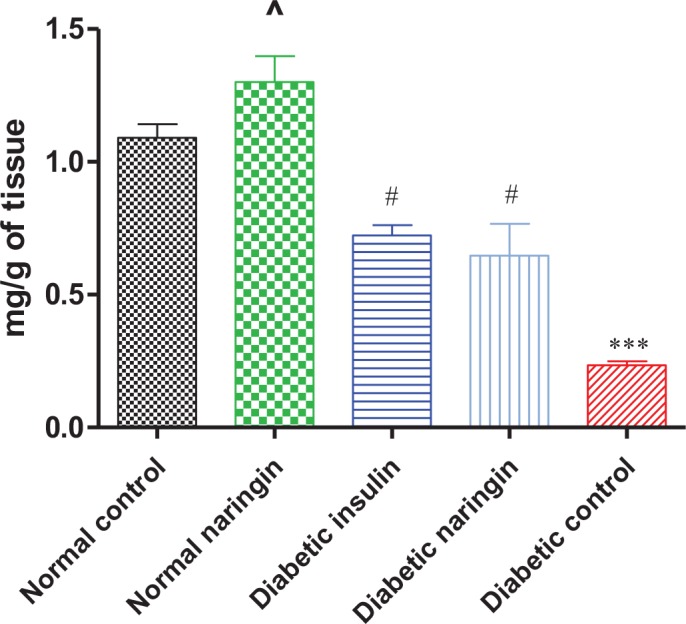
Glycogen levels in homogenised hepatic tissue. (***p< 0.0001, ^p< 0.05 compared to normal control, #p<0.05 compared to STZ only-treated rats, respectively).

Hepatic glycogen concentrations were significantly (p<0.0001) reduced in STZ-treated compared to the control group. Treatment with insulin or naringin significantly (p<0.05) increased hepatic glycogen content compared to the STZ only-treated group, respectively. Naringin treatment of normal rats caused significant (p<0.05) elevation of hepatic glycogen content compared to the normal control (Fig 7).

### Plasma Lipid Peroxidation (TBARS Assay)

**Fig 8:**
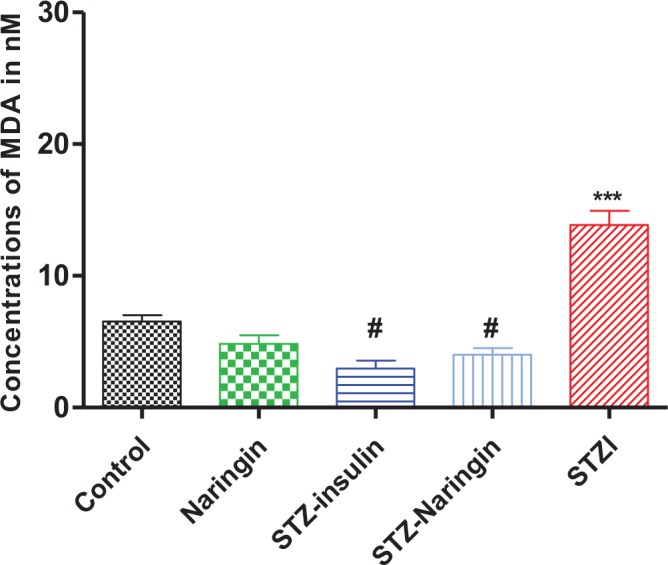
Plasma concentrations of MDA measured as a marker for lipid peroxidation. (***p<0.001 compared to normal control, #p<0.0001 compared to STZ only-treated group).

Plasma MDA concentrations in STZ only-treated group were significantly (p<0.0001) elevated compared to the normal control group. Naringin or insulin significantly (p<0.0001) reduced plasma MDA concentrations in STZ-treated rats compared to the STZ only treated group, respectively (Fig 8).

### Serum β-Hydroxybutyrate (3-HB) Levels

**Fig 9:**
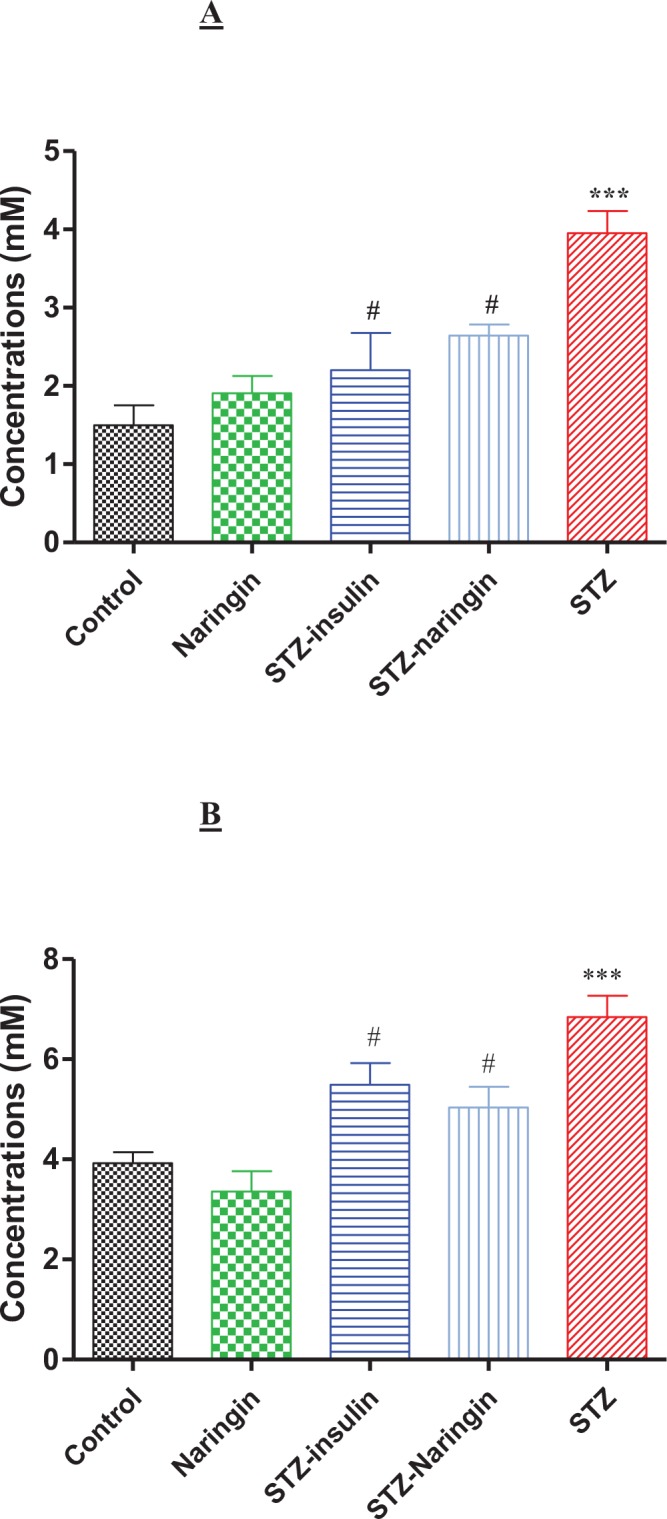
**A**; Serum 3-β-hydroxybutyrate (3HB) levels. (***p<0.0001 compared to normal untreated control, #p<0.05 compared to STZ only-treated rats) and **B**; Serum acetoacetate (AcAc) levels. (***p<0.0001 compared to normal control, #p<0.05 compared to STZ only-treated group).

Serum β-hydroxybutyrate (3-HB) levels were significantly (p<0.001) elevated in the STZ only-treated compared to the normal control group. Treatment with insulin or naringin significantly (p<0.05) reduced the levels of 3-HB compared to the STZ only-treated group, respectively (Fig 9A).

### Serum Acetoacetate (AcAc) Levels

Serum acetoacetate (AcAc) levels were significantly (p<0.0001) elevated in the STZ only-treated compared to normal control group. However, naringin significantly (p<0.05) reduced plasma AcAc levels in STZ-treated compared to the STZ only-treated rats (Fig 9B).

### Serum Electrolyte and Blood pH

Serum sodium, chloride and bicarbonate and blood pH were significantly (p<0.01, p<0.0001, p<0.0001 and p<0.05, respectively) reduced, while potassium was significantly (p<0.05) elevated in the STZ only-treated compared to the normal control group. Insulin significantly (p<0.05) increased sodium and reduced potassium in the STZ-treated compared to the STZ only-treated group, respectively. Naringin or insulin significantly (p<0.05) increased plasma bicarbonate and blood pH in STZ-treated compared to the STZ only-treated diabetic rats, respectively (Table 2).

**Table 2:** Serum electrolytes and blood pH. (^***^p<0.0001, ^**^p<0.01 and ^*^p<0.05 compared to the normal control group, ^#^p<0.0001, ^@^p<0.01 and ^&^p<0.05 compared to the STZ only-treated group).

	Normal control	Normal naringin	STZ-insulin	STZ-Naringin	STZ
Sodium (mM)	142.70±0.56	142.80±0.37	140.00±1.00**&**	132.20±1.50	135.00±1.79**
Potassium (mM)	6.42±0.30	7.120±0.59	5.87±0.12**&**	7.34±0.59	7.56±0.46*
Chloride (mM)	103.80±0.89	103.00±0.63	100.30±1.33	96.20±2.40	96.40±1.36***
Bicarbonate (mM)	24.77±0.76	24.74±1.04	21.18±0.69 **#**	17.63±0.64 **@**	13.10±0.54 ***
Blood pH	7.4±0.24	7.33±0.20	7.33±0.17**&**	7.4.00±0.31**@**	6.64±0.30**

### Anion Gap (AG)

**Fig 10:**
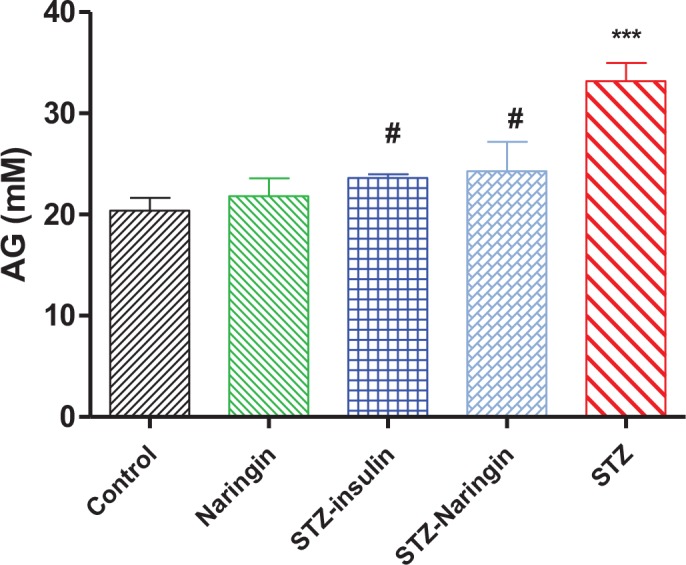
Anion gap (AG) calculated from serum electrolytes as previously described [11]. (^***^p< 0.0001 compared to normal control group, ^#^p< 0.05 compared to the STZ only-treated group).

Calculated AG was significantly (p<0.0001) increased in the STZ only-treated group compared to the normal control group. Naringin or insulin treatment significantly (p<0.05) decreased the serum AG in the STZ-treated groups compared to the STZ only-treated group, respectively (Fig 10).

## Discussion

This study was based on a typical type 1 diabetes model created with STZ which selectively and irreversibly completely destroys the pancreatic β-cells by oxidative damage. Interestingly, naringin treatment of normal rats was associated with relative increase in FPI concentrations (Fig 6). Considering that insulin treatment similarly increased FPI in diabetic animals, we speculate that the antioxidant effects of naringin reduced oxidative stress in the pancreatic β-cell mitochondria leading to increased ATP synthesis and subsequent insulin secretion. By its anabolic effects, insulin similarly to naringin, boosted residual anti-oxidant capacity of the β-cells leading to increased insulin secretion. This could have limited oxidative damage of STZ to allow the β-cells to retain some residual activity. Potential insulinotropic effects of naringin have only recently been demonstrated [22].

STZ-treated rats exhibited significant weight loss compared to controls (Fig 1). It may be argued that diabetes conferred hypophagic state since we did not measure food intake but it should also be considered that loss of body mass is a well known phenomenon in type 1 diabetes irrespective of food consumption rates [3, 13, 15]. That naringin, like insulin significantly attenuated weight loss in diabetic rats further suggests that, weight loss in diabetic rats was due to lipid and protein catabolism (associated with insulin deficiency) which were inhibited by both naringin and insulin, respectively. Hence we speculate that antioxidant effects of naringin reduced oxidative stress and free radicals which are known to provoke the release of catabolic insulin counter-regulatory hormones [9].

Polydipsia and polyuria were also observed in STZ-treated animals compared to the normal control group (Figs 2 and 3). Hyperglycemia in DKA leads to increased extracellular osmolality which causes dehydration. As a compensatory mechanism, increased osmolality activates hypothalamic osmoreceptors causing the release of antidiuretic hormone (ADH) which corrects the hyperosmolar state [23]. Failure of this mechanism leads to activation of thirst leading to the increased water intake. Insulin reversed both polyuria and polydipsia in STZ-treated rats. However, naringin treatment reversed only polydipsia but not polyuria suggesting that naringin inhibited thirst activation but had no anti-diuretic effects either in the normal or STZ-treated rats.

Unlike insulin, naringin improved neither FBG nor glucose intolerance in STZ-treated rats (Fig 4). We previously reported that naringin does not ameliorate hyperglycemia in diabetic type 1 rats [13]). This is contrary to other studies that have reported hypoglycemic effects of naringin and its aglycone naringenin to be mediated by suppression of hepatic expression of key gluconeogenic enzymes, phosphoenolpyruvate carboxykinase (PEPCK) and glucose-6-phosphatase (G6Pase) [13–15, 17, 24]. We concur with these observations but opine that these studies were done in simulated type 2 diabetes models where there was insulin resistance but not absolute deficiency. That insulin treatment significantly improved fasting blood glucose and plasma insulin, respectively suggests that by its anabolic effects insulin could have promoted modest pancreatic β-cell recovery in STZ- treated animals, enhanced residual insulin secretory capacity or both (Figs 4, 5B and 6). Glucose tolerance was similar between naringin-treated and control rats but calculated AUC suggested that STZ-treated rats were significantly glucose intolerant compared to normal controls (Fig 5). GTT was conducted with intraperitoneal injections of 3.0 mg/kg body weight of glucose in normal saline and even though we could not measure concurrent plasma insulin response, our results suggest that STZ-treated rats were already in hyperosmolar state considering the high fasting blood glucose concentrations (Figs 4 and 5B). Since insulin treatment was withheld during GTT, it is therefore not surprising that glucose tolerance did not improve in the insulin-treated group compared to STZ only-treated group.

Liver glycogen content was significantly reduced in STZ only-treated rats compared to controls but this was significantly reversed by either naringin or insulin treatment (Fig 7). This suggests that in the absence of insulin, glucagon and other insulin counter-regulatory hormones promoted gluconeogenesis and glycogenolysis which reduced glycogen storage in the liver in STZ-treated rats but this was inhibited by either naringin or insulin treatment. Naringin treatment of normal rats interestingly significantly increased hepatic glycogen content compared the normal controls (Fig 7). Insulin is known to activate glycogen storage by increasing expression of glycogen synthase which catalyses the rate-limiting step in glycogen synthesis [25]. Even though our study did not investigate the direct effects of naringin on hepatic expression of glycogen synthase, previous studies have shown that naringin or its aglycone naringenin suppresses PEPCK and G6Pase activities via activation of AMP-activated protein kinase (AMPK) [26, 27]. It is therefore likely that in our study, naringin failed to exert hypoglycemic effects due to insulin deficiency and hence argue that naringin has metformin-like effects.

Oxidative stress causes lipid peroxidation due to increased Reactive Oxygen Species (ROS) leading to overproduction of MDA, which is used as a biomarker [28, 29]. Lipid peroxidation was significantly elevated in the STZ-treated compared to the control group but was significantly reversed by naringin treatment (Fig 8). Oxidative stress stimulates production of pro-inflammatory cytokines which cause the release of insulin counter-regulatory hormones that are known to promote ketoacidosis [3]. Therefore, the antioxidant effects of naringin either directly or through enhancing antioxidant defence systems directly or by augmenting insulin secretion reversed ketoacidosis. This is demonstrated by the significantly reduced plasma 3-HB and AcAc in diabetic rats that were treated with naringin compared to the untreated diabetic group (Fig 9A and 9B). Insulin significantly reduced plasma 3-HB compared to untreated diabetic animals suggesting that naringin like insulin could be having a direct inhibitory effect on hydroxymethyl glutayryl Coenzyme A (HMGCoA) synthase, which catalyses the rate-limiting step in ketogenesis [9].

Jung et al., [25] previously showed that naringin suppresses plasma carnitine palmitoyl-O-transferase (CPT) which is involved in the transport of free fatty acids (FFA) across the mitochondrial membrane for β-oxidation and ketogenesis. Therefore, with reduced CPT levels, there is reduced ketogenesis due to decreased supply of FFA. Increased ketogenesis observed in diabetes is associated with overproduction and accumulation of ketone bodies which are strong organic anions. These dissociate freely producing hydrogen ions which bind and overwhelm serum bicarbonate buffering capacity eventually leading to metabolic acidosis in diabetes. However, naringin significantly improved plasma bicarbonate and blood pH levels, compared to STZ only-treated group (Table 2). Subsequently acid-base imbalance marked by increased AG was significantly reversed by naringin rats compared to STZ only-treated group (Fig 10). However, unlike insulin, naringin did not correct electrolyte imbalance in diabetic animals suggesting that like with insulin treatment, fluid replacement therapy should precede naringin supplementation were it to be used clinically. These observations confirm that naringin reversed metabolic acidosis associated with DKA albeit in experimental animal model. We recently reported antidiabetic effects of grapefruit juice in experimental animal studies [30]. Naringin is the main flavonoid in grapefruit juice and therefore it seems plausible to infer that some of the antidiabetic effects of grapefruit juice could be attributed to naringin in addition to other as yet unidentified bioactive chemical ingredients. However, naringin on its own appears in this study to be associated with favourable metabolic end-points in type 1 diabetes. There is no known toxicity of naringin both to humans or experimental laboratory animals [31, 32] and we used pharmacologically effective 50 mg/kg body of naringin [13, 33, 34] owing to its poor solubility in water which was dose limiting by oral gavage. Considering the failure of known endogenous antioxidants like vitamins E and C to exert positive pharmacological effects on degenerative metabolic diseases in large scale clinical trials [32], our results presented here suggest that citrus-fruit-derived flavanoids like naringin could be viable cheaper alternatives.

## Conclusion

Although naringin did not improve glucose intolerance, it was associated with reversal of weight loss, improved glycogen storage and insulin secretion in diabetic rats. However, naringin abrogated metabolic acidosis suggesting a role in the management of DKA. These actions of naringin appear to be mediated in part by its powerful antioxidant affects. Our findings therefore suggest that naringin as a nutritional supplement could be protective against ketonemia in type 1 diabetes patients and may be used as adjunct therapy pending further clinical studies.
